# Peripheral blood test provides a practical method for glioma evaluation and prognosis prediction

**DOI:** 10.1111/cns.13120

**Published:** 2019-03-26

**Authors:** Zhi‐Liang Wang, Chuan‐Bao Zhang, Yu‐Qing Liu, Zheng Wang, Tao Jiang

**Affiliations:** ^1^ Beijing Neurosurgical Institute, Capital Medical University Beijing China; ^2^ Department of Neurosurgery Beijing Tiantan Hospital, Capital Medical University Beijing China; ^3^ China National Clinical Research Center for Neurological Diseases Beijing China; ^4^ Center of Brain Tumor Beijing Institute for Brain Disorders Beijing China

**Keywords:** glioma, molecular markers, NLR, prognosis

## Abstract

**Objective:**

To investigate the relationship between tumor characteristics and the preoperative counts of immune cells in peripheral blood test in glioma patients.

**Methods:**

We included 260 WHO grades II‐IV patients who had preoperative peripheral blood test result from Sanbo hospital as training set. The 66 patients from Tiantan hospital was obtained for validation. RNA sequencing data from CGGA and TCGA datasets were used to evaluate the features of neutrophil subtype and lymphocyte subtype in glioma.

**Results:**

We revealed that the count of preoperative lymphocytes, eosinophils and neutrophils were associated with glioma grades. Neutrophil‐to‐lymphocyte ratio (NLR) <3.2 was associated with better prognosis, whereas increased NLR was strongly corresponding with a poor prognosis. Lymphocyte type glioma patients demonstrated a positive correlation with isocitrate dehydrogenase (IDH) mutation and lower grade. IDH mutant glioma contained a higher proportion of tumor‐infiltrating lymphocytes than IDH wild‐type glioma. The immune subtype (neutrophil subtype and lymphocyte subtype) was an independent prognostic factor in glioma.

**Conclusion:**

Our data demonstrated that NLR was an important prognostic factor in glioma. We classified that the immune subtype of glioma may contribute to a better understanding of disease pathogenesis and lead to the identification of new therapeutic targets for glioma patients.

## 1 INTRODUCTION

Glioma is the most common malignant primary tumor in central nervous system (CNS) in adults.^1^ The severity of gliomas is further distinguished by malignant grades (I to IV) according to features of cellular atypia, cell proliferation, angiogenesis, and necrosis.^2^ Despite recent advances in cancer diagnosis and treatment, the clinical efficiency and outcomes of glioma patients have not improved significantly. Glioblastoma remains one of the hardest cancers for treatment in clinical oncology.^3^ Previously, due to the presence of the blood‐brain barrier (BBB) and the absence of a classical lymphatic drainage system, the theory that brain was an immune‐privilege organ was widely accepted.^4^ Nowadays, inflammation has been identified to be a hallmark of cancer^5^ and the critical role of inflammation components in glioma was a well‐established concept.^6^, ^7^ Macrophages, T lymphocytes, and neutrophils were the important infiltrative inflammation cells in glioma microenvironment which could suppress or promote tumor progression.^8^, ^9^, ^10^, ^11^

However, whether the inflammation induced by glioma could trigger body's innate immune system was unclear. Routine blood test, low cost and easy to measure, is a regular examination for patients which give us information reflecting innate immune system. Here, we attempted to explore the relationship between tumor characteristics and routine blood test parameters in glioma. The first‐time preoperative routine blood test, RNA sequencing data, and clinicopathological information of glioma patients were included in this study. We found that patients in highly neutrophil‐to‐lymphocyte ratio (NLR) group significantly had a shorter overall survival. Correspondingly, neutrophil subtype glioma patients significantly had a poor prognostic than lymphocyte subtype glioma patients. The integrated analysis of routine blood test and RNA sequencing data may provide a new perspective for immunotherapy in the future.

## 2 MATERIALS AND METHODS

### 2.1 Study population

We collected 260 glioma patients (69 grade II, 52 grade III, 139 grade IV) with isocitrate dehydrogenase (IDH) information from Sanbo Brain Hospital, Capital Medical University from 2009 to 2015 as training cohort. The validation cohort included 66 glioma patients (9 grade II, 19 grade III, 38 grade IV) from Beijing Tiantan Hospital. The first‐time preoperative peripheral blood cells data of glioma samples were available. These patients did not catch acute conditions like bacterial or viral infections or drug treatments which might affect human body immune system from consulting hospital case document. This study was approved by the institutional review boards of all hospitals involved in the study, and written‐informed consent was obtained from all patients.

RNA expression profiling of human immune cell subsets was downloaded from GEO datasets (https://www.ncbi.nlm.nih.gov/geo/query/acc.cgi?acc=GSE28492). The neutrophil and lymphocyte signatures were defined as significantly differently expressed genes between the neutrophil cell lines and lymphocyte cell lines. RNA sequencing data, molecular pathological data, and clinical information of 325 glioma patients were obtained from Chinese Glioma Genome Atlas (CGGA) database (http://www.cgga.org.cn) and 699 glioma patients from the Cancer Genome Atlas (TCGA) database^12^ (http://cancergenome.nih.gov). The RNA sequencing data were log2 transformed before the following analysis.

### 2.2 Biomarker detection

Genomic DNA was isolated from frozen tumor tissues or paraffin‐embedded samples by using the QIAamp DNA Mini Kit (Qiagen). Pyrosequencing analysis was carried out by Gene Tech (Shanghai) Company Limited. The mutational status of IDH1/2 and the methylation status of the MGMT promoter were determined using pyrosequencing or Sanger sequencing as previously reported.^13^

### 2.3 Immunohistochemistry

T lymphocytes were distinguished by antibody against CD3 (ZA‐0503, Zhongshan‐BIO, 1:100 dilution). To precisely quantify T lymphocytes in glioma, under high magnification (×400) microscopic fields, we counted more than 1000 tumor cells, of which positively stained cells were reckoned in as well. The ratio of T lymphocytes in tumor was defined as the portion of positively stained cells against total counted cells. The difference was assessed by Mann‐Whitney test.

### 2.4 Statistical analysis

Unpaired *t* test or Mann‐Whitney test were used for comparison of two groups. And the samples distribution was evaluated by Chi‐squared test. PFS was defined as the time of surgery until radiographic progression (the appearance of a new lesion or an increase in tumor size of >25%).^14^ Overall survival (OS) was defined as time interval from histologic diagnosis to death. Patients who were still alive or lost to follow‐up were censored at last follow‐up. OS and PFS were estimated with Kaplan‐Meier method and compared with log‐rank method by using the GraphPad Prism version 6.0 statistical software. Univariate and multivariate cox proportional hazard model were evaluated using coxph function from survival package. A two‐sided *P* value < 0.05 was regarded as significant.

## 3 RESULTS

### 3.1 Peripheral blood immune cells in WHO grade II‐IV primary glioma patients

**Figure 1:**
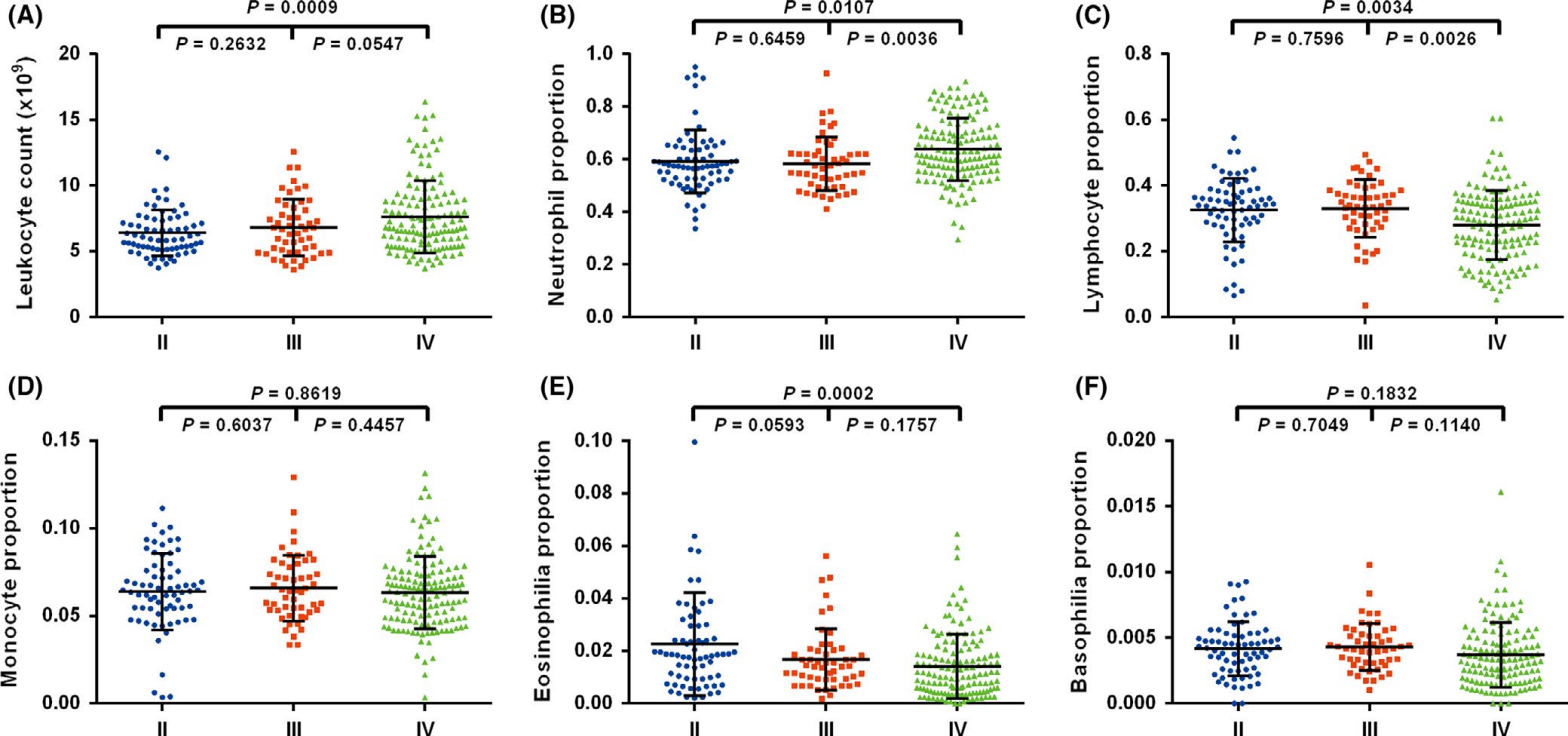
Comparison of preoperative peripheral blood content among three grades gliomas. A, Leukocytes count was different between grade II (N = 69) and IV samples (N = 139, *P* = 0.0009). B and C, neutrophils, and lymphocytes proportion were different between grade III (N = 52) and grade IV, grade II, and grade IV, respectively. D and E and F, except for eosinophils between grade II and grade IV (*P* = 0.0002), monocytes, eosinophils, and basophils showed no difference among three grades gliomas (*P* > 0.05)

We assessed the count of leukocytes, the count and the proportion of five leukocyte subtypes (neutrophil, lymphocyte, monocyte, eosinophil, and basophil) in peripheral blood according to WHO grades. As shown in Figure 1 and Figure S1, grade IV patients demonstrated a significant increase in the count of white cells compared with that observed in grade II (*P* = 0.0009). Neutrophils showed similar trend. The proportion and count of neutrophils in grade IV were significantly higher than grade II (*P* = 0.0107, *P* = 0.0007) and grade III patients (*P* = 0.0036, *P* = 0.0168). However, the lymphocytes proportion exhibited an inverse tendency, which showed a significant decrease in grade IV than in grade II (*P* = 0.0034) and grade III patients (*P* = 0.0026). There was no statistically significant difference was observed among tumor grades of lymphocyte counts. The proportion and counts of eosinophils were obviously higher in grade II than grade IV patients (*P* = 0.0002, *P* = 0.0007), and its count was also significantly increased in grade II than grade III (*P* = 0.0496).

### 3.2 The prognostic value of preoperative NLR in primary glioma patients

**Figure 2:**
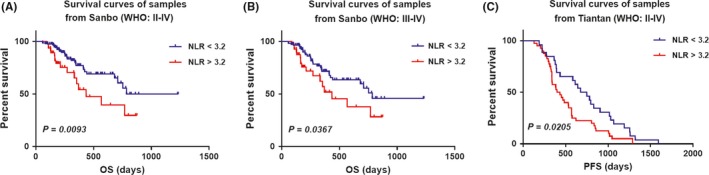
Prognostic value of neutrophil‐to‐lymphocyte ratio (NLR) in Sanbo (group1, N = 260) and Tiantan (group2, N = 66) cohorts. A, Patients (grade II‐IV from Sanbo Brain Hospital), B, High grade (grade III‐IV) patients (from Sanbo Brain Hospital) and C, Patients (grade II‐IV from Tiantan Hospital) with higher NLR showed worse prognosis (A, NLR <3.2 group, N = 162, median survival: 784 days; NLR >3.2 group, N = 46, median survival: 436 days; *P* = 0.0093; B, NLR <3.2 group, N = 115, median survival: 784 days; NLR >3.2 group, N = 40, median survival: 436 days, *P* = 0.0367; C, NLR <3.2 group, N = 26, median survival: 713.5 days; NLR >3.2 group, N = 40, median survival: 416.5 days, *P* = 0.0205)

Neutrophil‐to‐lymphocyte ratio (NLR) is a marker of systemic inflammatory response which exhibited the prognostic and predictive values in various cancers, including colon, prostate^15^ and bladder cancer.^16^ Here, we investigated the correlation between NLR and overall survival in glioma. NLR was analyzed as a continuous variable. We assessed optimal cutoff dependent on log‐rank testing from 3 to 4.5 (Table S1) and the value of 3.2 with minimum *P*‐value (*P* = 0.0093) was selected as the cutoff point to provide prognostic information. Patients within NLR <3.2 group (N = 160, median survival: 784 days) were significantly associated with increased overall survival relative to those samples with NLR >3.2 (N = 48, median survival: 436 days) (*P* = 0.0093, Figure 2A) in the glioma patients from Sanbo hospital. Moreover, the patients in higher grade (WHO grade III‐IV) glioma also demonstrated the similar result. Patients in NLR <3.2 group (N = 115, median survival: 784 days) had a better outcome than in NLR >3.2 group (N = 40, median survival: 436 days) (*P* = 0.0367, Figure 2B).

This survival association was validated in patients from Tiantan hospital(N = 66), and similar result was detected. The median progression‐free survival of patients in NLR <3.2 group was 713.5 days, and in NLR >3.2 group was 416.5 days (*P* = 0.0205, Figure 2C). Overall, these data indicated that an elevated NLR significantly correlated with a worse survival outcome.

### 3.3 The relationship between NLR and molecular markers

IDH mutation, somatic mutation in isocitrate dehydrogenase one and two gene, occurs at high frequency in gliomas and is a prognostic factor in glioma patients. IDH gene mutation was demonstrated with younger, secondary GBMs (sGBMs), and better outcomes.^17^ There was a significant different in NLR between IDH mutation group and IDH wild‐type group (*P* = 0.0347, Figure S2A). Patients with NLR <3.2 were more likely to be IDH mutated.

MGMT promoter methylation is a predictive marker for TMZ chemotherapy.^18^ We also tested the MGMT promoter methylation status in 190 out of 260 patients by pyrosequencing. There was not significant difference between MGMT promoter methylated group and unmethylated group (*P* = 0.5434, unpaired *t* test, Figure S2B).

### 3.4 Generation of neutrophil and lymphocyte expression signatures

**Figure 3:**
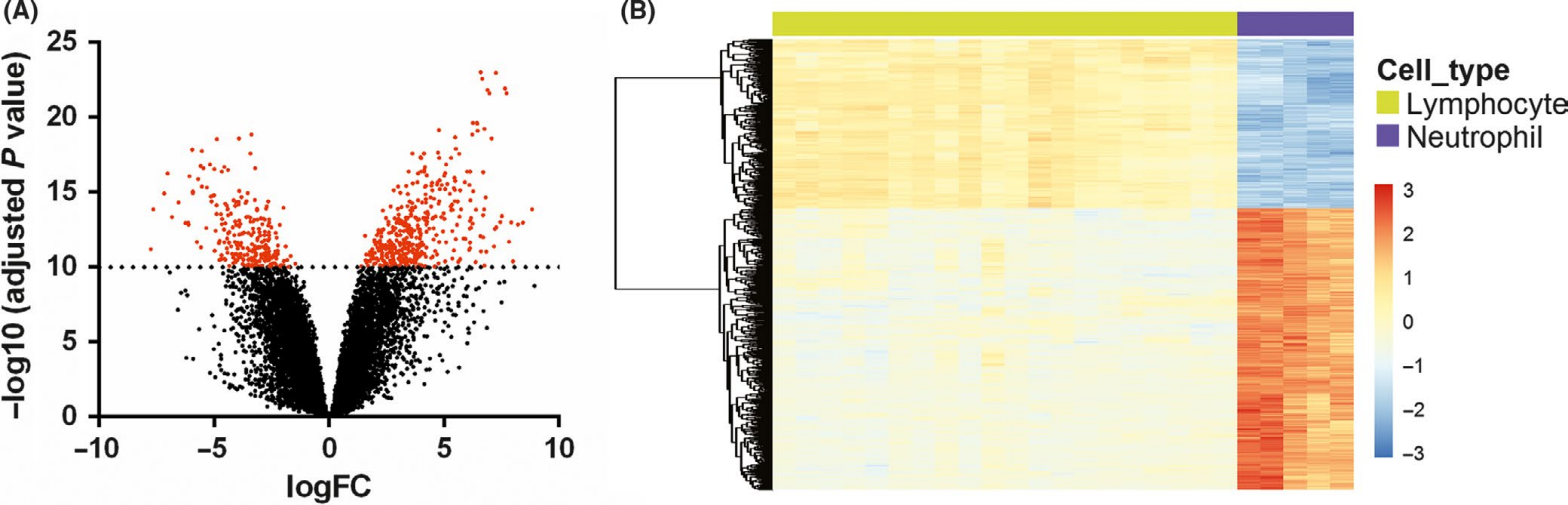
Generation of lymphocyte and neutrophil expression signatures. A, Differential expression analysis between RNA expression profiling of lymphocytes and neutrophils. Red dots, significantly differentially expressed probes (adjusted *P* value < 1e‐10, 661 probes, 520 genes, Table S1). B, expression patterns of the 661 probes

The NLR parameters based on pre‐operation peripheral blood test showed prognostic value in glioma patients. However, the clinical value of neutrophil and lymphocyte signatures from RNA sequencing data in glioma were not investigated comprehensively. To systematically evaluate the relationship the neutrophil and lymphocyte signatures in glioma, we downloaded RNA expression profiling of human immune cell subsets (GSE28492, five neutrophils samples and 20 lymphocytes samples) from GEO dataset. After log2 transformation, *t* test with Benjamini & Hochberg adjustment was used for differential expression analysis. The lymphocyte and neutrophil signatures were defined as genes differently expressed between lymphocyte and neutrophil (adjusted *P* value < 1e‐10, Table S3). Finally, 258 probes (229 genes) highly expressed in lymphocytes and 403 probes (291 genes) highly expressed in neutrophils were defined as cell‐type specific signatures (Figure 3A, B).

### 3.5 Clinicopathological features of lymphocyte and neutrophil type glioma patients

To elucidate the clinical and genetic features of lymphocyte and neutrophil type gliomas, we clustered glioma samples from CGGA and TCGA dataset by *k*‐means (*k* = 2) method. Tumor‐associated macrophage (TAM), playing crucial roles in glioma progression, represent about more than a half of immune cells in tumor mass.^19^, ^20^ Here, we used transcriptome data to generate enrichment score with macrophage signatures and depicted the characteristics of macrophage in lymphocyte and neutrophil type gliomas.

**Figure 4:**
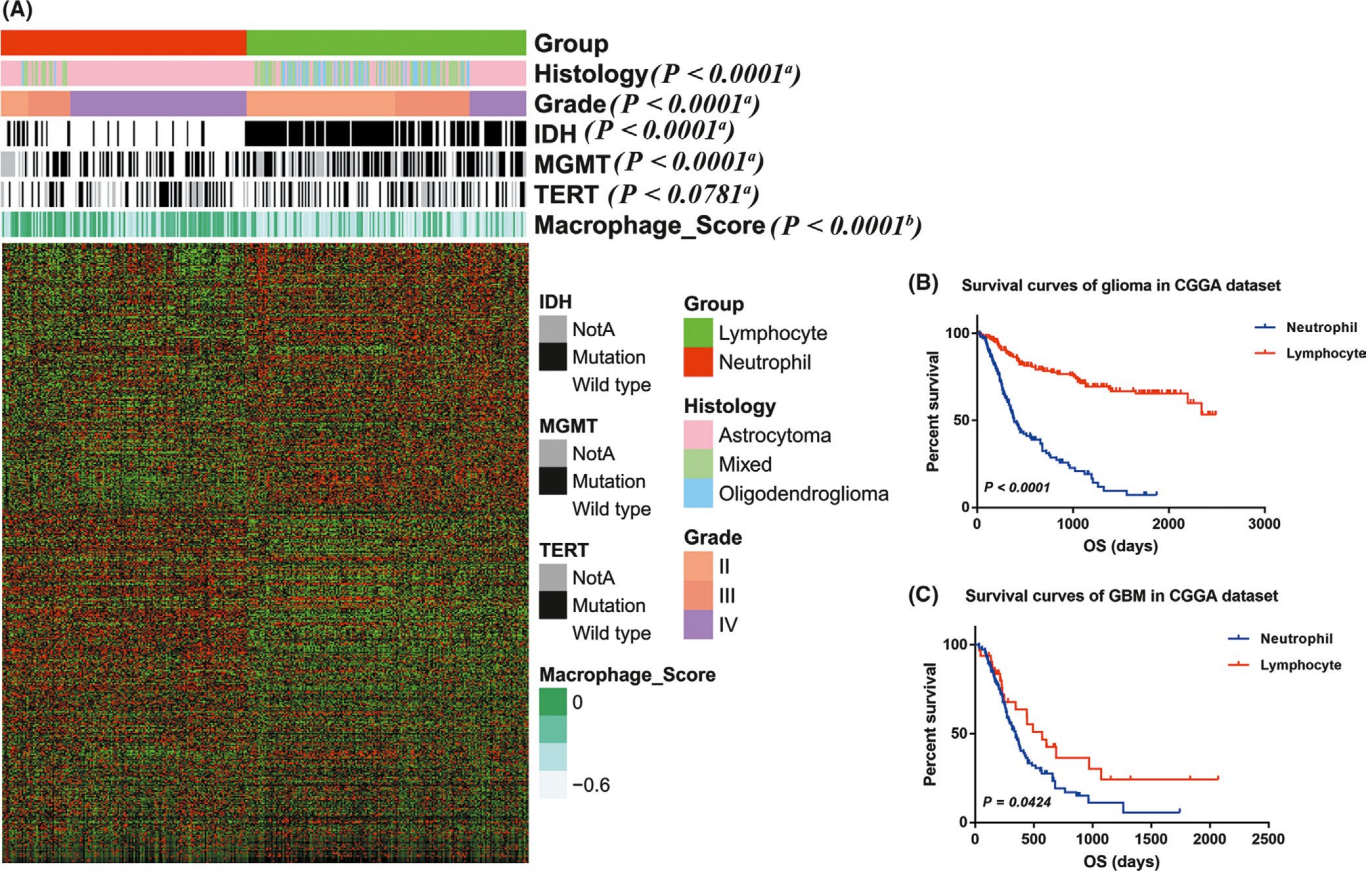
Classification of CGGA glioma patients based on lymphocyte and neutrophil signatures. A, RNAseq data of 325 glioma samples from CGGA dataset were clustered based on the signatures. In lymphocyte type (with high expression of lymphocyte signatures), grade II and III, oligodendroglioma and oligoastrocytoma, and IDH mutant samples were enriched, while GBM and IDH wild‐type samples were more likely to be clustered into neutrophil type. B, C, lymphocyte type patients showed more favorable prognosis than neutrophil ones (all patients, *P* < 0.0001; GBM patients, *P* = 0.0424). The relationship between neutrophil subtype and lymphosubtype and patients' characteristics was evaluated (a, Chi‐square test. b, Student's *t* test)

In CGGA dataset, lymphocyte type gliomas (N = 173) were high expression of lymphocyte signatures (Figure 4A). The majority of grade II and III (138/181, 76%), oligocytic (oligodendroglioma and oligoastrocytoma, 97/114, 85%) and IDH mutant (143/167, 86%) tumors were enriched in this subtype, while MGMT promotor methylated conferred in lymphocyte type glioma. Meanwhile, 76% (109/144) GBM and 81% (128/158) IDH wild‐type tumors were clustered in neutrophil subtype. Higher macrophage enrichment scores were more likely to belong to neutrophil subtype glioma. Lymphocyte type glioma patients (median survival not reached, median follow‐up 1047 days) had significant longer survival than neutrophil ones (median survival 386 days, *P* < 0.0001, Figure 4B). For GBM patients, lymphocyte GBMs (median survival 572 days) also showed superior survival than neutrophil ones (median survival 345 days, *P* < 0.05, Figure 4C).

**Figure 5:**
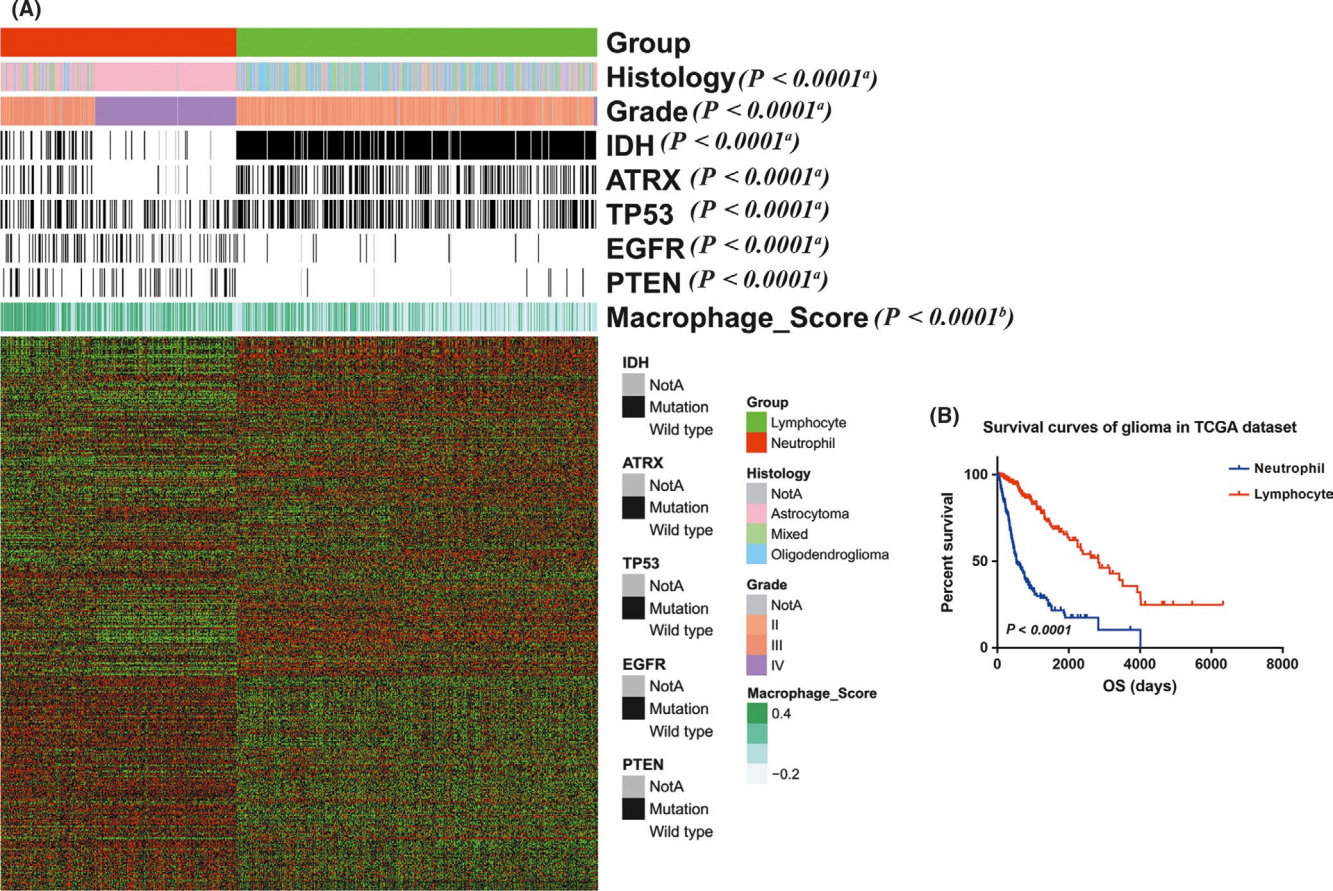
Validation of the classification based on lymphocyte and neutrophil signatures in TCGA dataset. A, RNAseq data of 699 glioma samples from TCGA dataset were clustered to validate the classification result. Similarly, Grade II and III, oligodendroglioma, IDH, and ATRX mutant samples were more likely to be clustered into lymphocyte type, while the others were in neutrophil type. B, the lymphocyte type glioma patients had more favorable prognosis than neutrophil ones (*P* < 0.0001). The relationship between neutrophil subtype and lymphosubtype and patients' characteristics was evaluated (a, Chi‐square test. b, Student's *t* test)

In TCGA dataset (Figure 5A), the same clustering approach was applied. Similar to the results in CGGA dataset, a vast majority of grade II and III (412/519, 79%), oligocytic (oligodendroglioma and oligoastrocytoma, 295/327, 90%), IDH (396/439, 90%) and ATRX (177/209, 85%) mutation tumors were enriched in this subtype, while 98% (164/168) GBM, 90% (227/251) IDH wild type, 86% (67/78) EGFR, and 89% (50/56) PTEN mutant tumors were clustered in neutrophil subtype. And macrophage enrichment scores were significantly higher in neutrophil subtype. The lymphocyte type glioma patients had more favorable prognosis (median survival 2875 days) than neutrophil ones (median survival 460 days, *P* < 0.0001, Figure 5B).

Furthermore, Cox regression analysis was performed additionally, verifying the independence clinical prognostic value of neutrophil and lymphocyte signature in glioma. In the above two datasets, in univariate cox analysis, it was showed that age, IDH status, 1p19q status and neutrophil and lymphocyte subtype were significantly associated with OS. In multivariate analysis, the immune subtype was also a significant factor after adjusting for the clinical factors mentioned above (Table 1 and Table S2).

**Table 1:** Cox hazard regression analyses of clinicopathologic factors and the neutrophil and lymphocyte subtypes for overall survival in CGGA (N = 325)

	Univariate cox model			Multivariate cox model		
	Hazard ratio	95%CI	*P* value	Hazard ratio	95%CI	*P* value
Male vs Female	1.18	0.84‐1.67	0.35			
Age ≥40 vs <40	1.61	1.13‐2.96	0.008	0.83	0.56‐1.24	0.37
IDH status MUT vs WT	0.23	0.16‐0.33	<0.0001	0.51	0.31‐0.81	0.005
1p19q intact vs codel	0.13	0.06‐0.27	<0.0001	0.25	0.12‐0.51	0.0001
Neutrophil subtype vs lymphocyte subtype	5.02	3.46‐7.29	<0.0001	2.62	1.62‐4.05	<0.0001

### 3.6 Lymphocyte proportion demonstrated a positive correlation with IDH mutation in glioma

**Figure 6:**
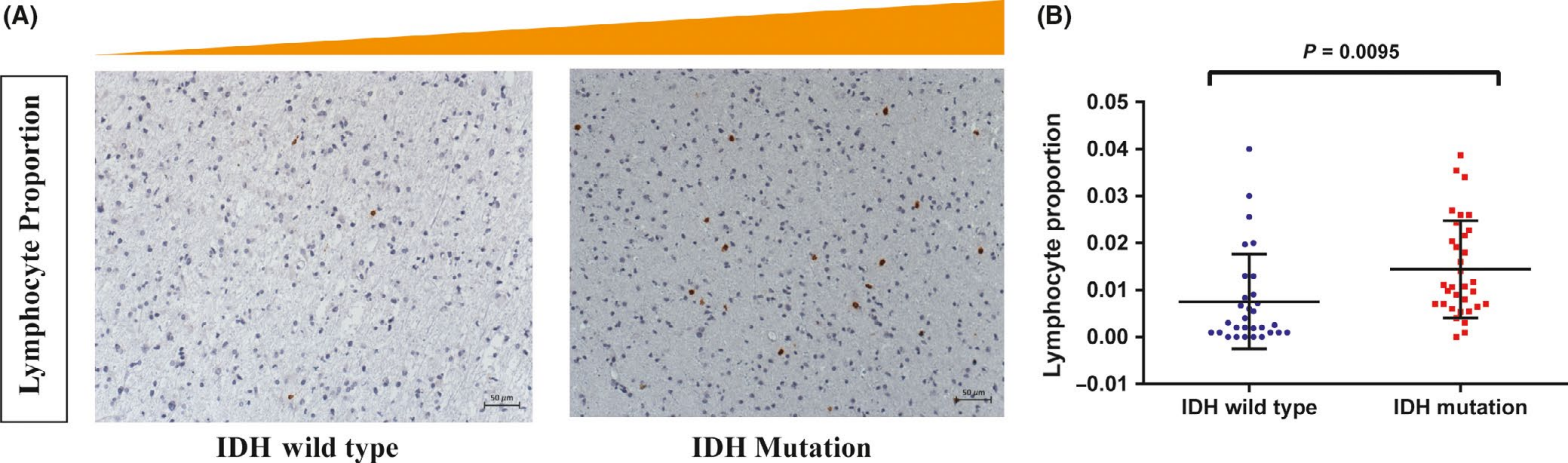
Validation of association between lymphocyte proportion and IDH mutation in an independent validation dataset (N = 68). A. Photographs of immunohistochemical staining of CD3 in IDH mutation and wild‐type groups of gliomas. Positive cells are stained brown. B. The lymphocyte proportion was higher in IDH mutant samples (*P* = 0.0095)

To further validate the relationship between lymphocytes and characteristics of gliomas, we evaluated the lymphocyte proportion and IDH mutation status in 68 glioma samples from Beijing Tiantan Hospital by Immunohistochemistry (IHC) staining. We quantified lymphocyte proportion using CD3 (T‐cell marker, the main lymphocyte in gliomas^21^, ^22^) immunostaining (Figure 6A). The proportion was defined as lymphocyte count divided by total tumor cells (>1000) in several 400× high cellular fields. The lymphocyte proportion ranged from 0 to 0.04 (median: 0.0075) in IDH wild‐type samples, and from 0 to 0.0387 (median: 0.0144) in IDH mutant samples (Figure 6B). IDH mutant samples (range: 0‐0.0387, median: 0.0144) showed significantly higher lymphocyte proportion than IDH wild‐type samples (range: 0‐0.04, median: 0.0075, *P* = 0.0095).

## 4 DISCUSSION

To our knowledge, this study was the first systematic study that comprehensively revealed the strong association between peripheral blood immune cells and tumor characteristics. Neutrophil and eosinophil belong to innate immune cells while lymphocyte belongs to adaptive immune cells.^23^ The lymphocyte is the surrogate of an impaired cell‐mediated immunity,^24^ while neutrophil is acknowledged as a response to systematic inflammation.^25^ The recruitment of neutrophil and lymphocyte play a critical component in the pathogenesis of gliomas.^11^ Our study demonstrated that the counts of lymphocytes were significantly higher at early stage of glioma and then decreased with tumor progression. The glioblastoma multiforme (WHO IV) possessed the highest extent of neutrophil infiltration and the lowest level of lymphocyte. Moreover, NLR was a prognostic marker in gliomas, which was consistent with findings in multiple other solid tumors.^26^ Using a cutoff NLR value of four, Raushan auezova et al^27^ reported on the associations of NLR with OS in 178 patients and Jing Zhang et al^28^ demonstrated that higher NLR was correlated with shorter OS. In our study, we found that the OS of patients in NLR >3.2 group was significantly shorter than in NLR <3.2 group. As shown in Table S1, pre‐operation NLR <4 and NLR <3.2 were all strongly associated with improved OS. And the difference between groups divided by NLR value of 3.2 was more statistically significant than divided by NLR value of four in our research.

Furthermore, in CGGA dataset, we divided glioma patients into two types with immune cell‐specific signatures (lymphocyte type and neutrophil type) to investigate the immune subtype of glioma. And, we validated our immune subtype classification system in TCGA dataset. Our immune subtype classification system effectively classified patients into lymphocyte and neutrophil subtype. The lymphocyte subgroup was characterized by good clinical outcome, younger age, low malignant behaviors, and extraordinary high IDH1 mutation. The neutrophil groups exhibited the opposite effect.

It has been reported that IDH mutation was a frequent genomic alteration in grade II and grade III gliomas but rare in primary glioblastoma.^17^ Tumors with IDH mutations had distinct genetic and clinical characteristics, and patients with such mutations had a better outcome than those with wild‐type IDH. We hypothesise that IDH mutation glioma has a more heterogeneous microenvironment that can affect the cytokine production which will affect the amount and function of lymphocyte and neutrophils. And the cytokines can contribute to further disease progression by interacting with the inflamed BBB endothelium, and for some cell types, entering the CNS by crossing vascular barriers. Our IHC results found that the extent of tumor infiltration lymphocyte was significantly higher in IDH mutant glioma than IDH wild‐type glioma. This phenomenon may potentially indicate that the tumor immune microenvironment of glioma differed in combination with their IDH status. However, the mechanism of the association of IDH mutation and the immunologic tumor microenvironment remained unclear.

We recognized some limitation in our research. First, the peripheral blood immune cells count and NLR can be affected easily, such as chronic diseases, local or systemic infection, previous history of infection (>3 months) and any medication that related to inflammatory condition of patients. Second, limited by the small patient number and the retrospective nature of our study, a prospective study with large number of patients is required.

## 5 CONCLUSIONS

In summary, our data showed that pre‐operation NLR was a readily available and effective prognostic factor in glioma patients. Furthermore, we classified the immune subtype of glioma with neutrophil and lymphocyte signatures, and we found that IDH mutant glioma contained a higher proportion of tumor‐infiltrating lymphocytes than IDH wild‐type glioma. Although the mechanisms need further studies, we provided a potential, easily accessible, and reliable method for glioma evaluation in clinical practice.

## CONFLICT OF INTEREST

The authors declare that the research was conducted in the absence of any commercial or financial relationships that could be construed as a potential conflict of interest.
