# Supplementary material for: Peripheral blood test provides a practical method for glioma evaluation and prognosis prediction
Source: CNS Neurosci Ther. 2019 Mar 26;25(8):876–83. doi: 10.1111/cns.13120 (PMC6630006; doi:10.1111/cns.13120)
Supplement: Supplementary file 1 [file CNS-25-876-s001.docx]

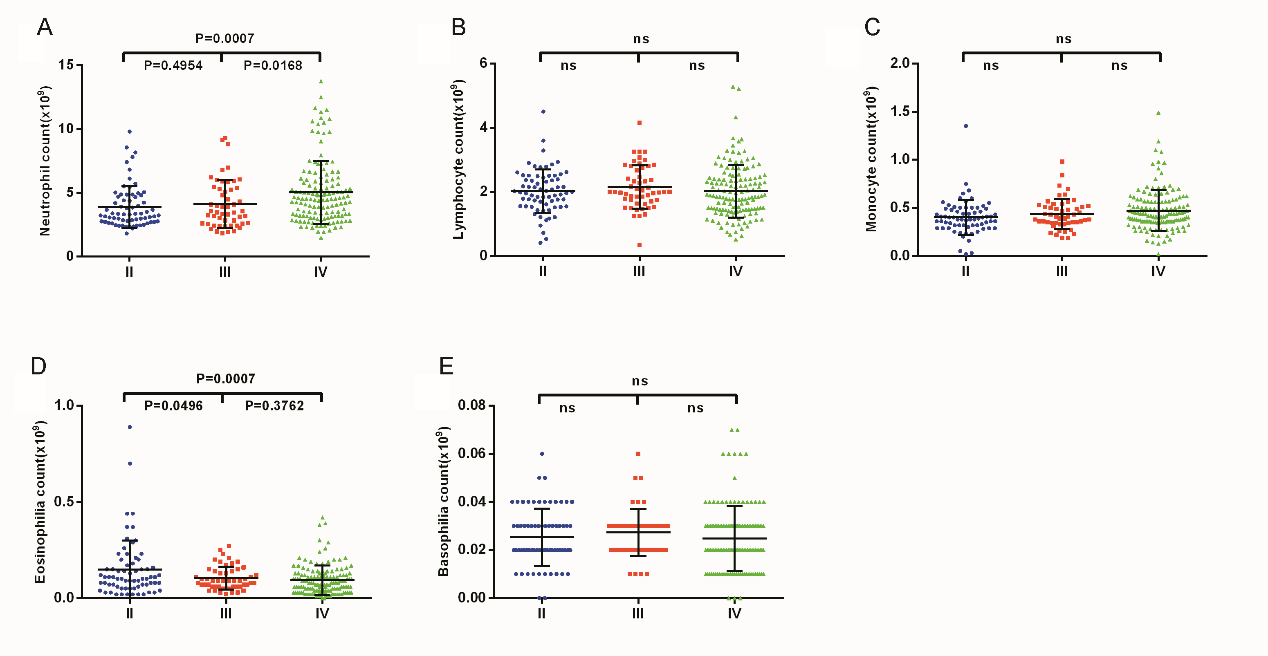
 Figure S1. Comparison of preoperative peripheral blood content among three grades gliomas. Neutrophils were different between grade II and IV, grade III and IV (A). There was no significant difference (ns) of lymphocytes, monocytes and basophils among the three grades (B, C, E). Eosinophils were different between grade II and IV, grade II and III (D).


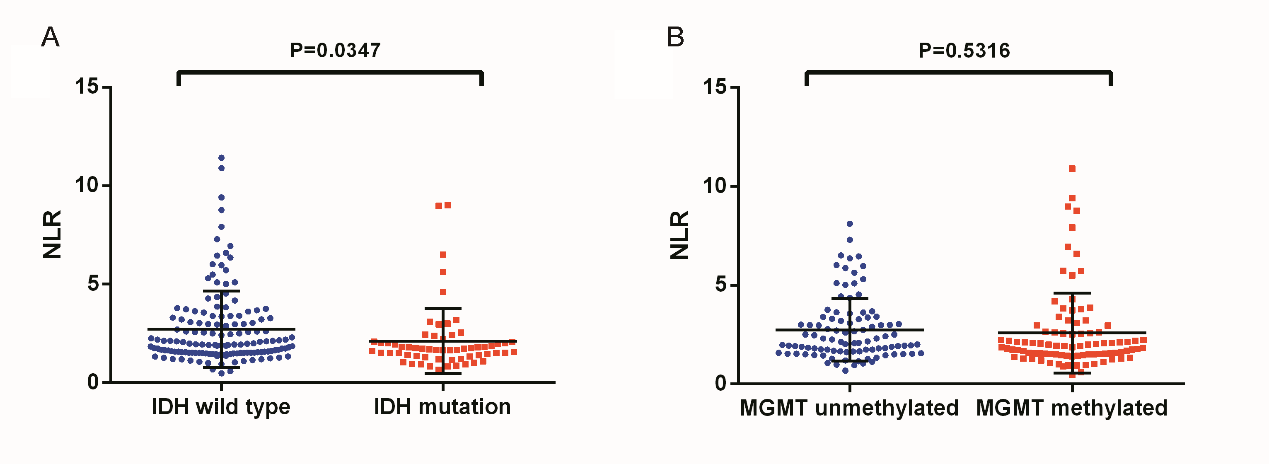


Figure S2. The relationship between NLR and molecular markers. A, NLR were higher in IDH wild type samples (p=0.0347). B, there was no significant difference between MGMT promoter methylated samples and unmethylated samples (p=0.5434).

Table S1. The log-rank testing results of cutoff values from 3 to 4.5

| Cutoff value | HR value | P.value |
| --- | --- | --- |
| 3 | 2.054015 | 0.012855 |
| 3.1 | 1.990531 | 0.018709 |
| **3.2** | **2.154935** | **0.009315** |
| 3.3 | 1.850985 | 0.044993 |
| 3.4 | 2.030782 | 0.022965 |
| 3.5 | 1.851337 | 0.054437 |
| 3.6 | 1.917345 | 0.041729 |
| 3.7 | 1.747786 | 0.090248 |
| 3.8 | 1.572766 | 0.199841 |
| 3.9 | 1.869156 | 0.075351 |
| **4** | **2.056493** | **0.039479** |
| 4.1 | 2.056493 | 0.039479 |
| 4.2 | 2.075241 | 0.036914 |
| 4.3 | 1.854523 | 0.091549 |
| 4.4 | 1.913111 | 0.075757 |
| 4.5 | 1.913111 | 0.075757 |

Table S2. Cox Hazard Regression Analyses of Clinicopathologic Factors and the Neutrophil and lymphocyte subtypes for Overall Survival in TCGA (N=699)

|  | **Univariate Cox Model** | | | **Multivariate Cox Model** | | |
| --- | --- | --- | --- | --- | --- | --- |
|  | **Hazard ratio** | **95%CI** | ***p* value** | **Hazard ratio** | **95%CI** | ***p* value** |
| **Male vs. Female** | **1.09** | **0.82-1.44** | **0.55** |  |  |  |
| **Age ≥40 vs. <40** | **5.31** | **3.92-7.19** | **<0.0001** | **2.38** | **1.69-3.35** | **<0.0001** |
| **IDH status MUT vs. WT** | **0.11** | **0.08-0.15** | **<0.0001** | **0.22** | **0.14-0.34** | **<0.0001** |
| **1p19q intact vs. codel** | **0.21** | **0.13-0.36** | **<0.0001** | **0.65** | **0.36-1.71** | **0.15** |
| **Neutrophil subtype vs. Lymphocyte subtype** | **4.81** | **3.50-6.61** | **<0.0001** | **1.74** | **1.19-2.54** | **0.0045** |

Table S3. Differential expression analysis between neutrophil and lymphocyte.

| ID | Gene.symbol | adj.P.Val | logFC (Lym/Neu) |
| --- | --- | --- | --- |
| 211372_s_at | IL1R2 | 9.84E-24 | 6.5965907 |
| 210119_at | KCNJ15 | 1.06E-23 | 7.2580491 |
| 221345_at | FFAR2 | 2.69E-23 | 6.6648488 |
| 204470_at | CXCL1 | 1.2E-22 | 7.6551867 |
| 211163_s_at | TNFRSF10C | 1.48E-22 | 6.8938587 |
| 205403_at | IL1R2 | 2.43E-22 | 7.7145696 |
| 229967_at | CMTM2 | 2.43E-22 | 6.9754199 |
| 206765_at | KCNJ2 | 2.31E-20 | 6.4258326 |
| 203691_at | PI3 | 2.37E-20 | 6.259065 |
| 225987_at | STEAP4 | 5.66E-20 | 6.7499134 |
| 214681_at | GK | 7.02E-20 | 4.7766586 |
| 203591_s_at | CSF3R | 7.63E-20 | 6.4514193 |
| 200017_at | RPS27A | 1.37E-19 | -3.3759406 |
| 41469_at | PI3 | 1.37E-19 | 6.2321153 |
| 218963_s_at | KRT23 | 2.14E-19 | 5.4855295 |
| 218203_at | ALG5 | 2.51E-19 | -3.9233333 |
| 206515_at | CYP4F3 | 2.6E-19 | 7.0642048 |
| 200725_x_at | RPL10 | 2.82E-19 | -4.8783601 |
| 220528_at | VNN3 | 1.24E-18 | 5.5050666 |
| 205040_at | ORM1 | 1.24E-18 | 5.5202599 |
| 201754_at | COX6C | 1.42E-18 | -5.9511092 |
| 200926_at | RPS23 | 1.77E-18 | -5.5338082 |
| 226064_s_at | DGAT2 | 2.16E-18 | 4.7353699 |
| 211307_s_at | FCAR | 2.51E-18 | 4.12906 |
| 201094_at | RPS29 | 2.56E-18 | -3.4156719 |
| 207674_at | FCAR | 2.7E-18 | 5.6554318 |
| 204308_s_at | TECPR2 | 4.47E-18 | 3.9718742 |
| 203435_s_at | MME | 4.97E-18 | 5.3080534 |
| 228648_at | LRG1 | 5E-18 | 4.7909176 |
| 228754_at | SLC6A6 | 5.16E-18 | 4.010332 |
| 212578_x_at | RPS17 | 1.36E-17 | -5.1789656 |
| 217838_s_at | EVL | 1.77E-17 | -5.5870218 |
| 207890_s_at | MMP25 | 2.05E-17 | 4.1613604 |
| 217802_s_at | NUCKS1 | 2.05E-17 | -4.5618109 |
| 217807_s_at | GLTSCR2 | 2.41E-17 | -5.4683758 |
| 200963_x_at | RPL31 | 2.41E-17 | -3.2246719 |
| 227261_at | KLF12 | 2.87E-17 | -4.9908258 |
| 211306_s_at | FCAR | 3.31E-17 | 4.1413058 |
| 209134_s_at | RPS6 | 3.31E-17 | -4.3985372 |
| 220005_at | P2RY13 | 4.06E-17 | 6.1312612 |
| 200081_s_at | RPS6 | 4.06E-17 | -4.6666475 |
| 212659_s_at | IL1RN | 4.06E-17 | 4.0016333 |
| 211668_s_at | PLAU | 4.06E-17 | 3.4630209 |
| 229228_at | CREB5 | 4.06E-17 | 6.0146599 |
| 202912_at | ADM | 4.43E-17 | 6.7033808 |
| 210772_at | FPR2 | 5.29E-17 | 5.0426631 |
| 211806_s_at | KCNJ15 | 5.55E-17 | 3.8740398 |
| 216834_at | RGS1 | 5.55E-17 | -7.027446 |
| 209179_s_at | MBOAT7 | 7.18E-17 | 4.2414219 |
| 221541_at | CRISPLD2 | 7.7E-17 | 5.0126997 |
| 209609_s_at | MRPL9 | 8.47E-17 | -6.0733851 |
| 211666_x_at | RPL3 | 1.32E-16 | -5.7035161 |
| 210233_at | IL1RAP | 1.6E-16 | 4.8080125 |
| 202888_s_at | ANPEP | 1.71E-16 | 5.4766088 |
| 216243_s_at | IL1RN | 2.09E-16 | 3.8364702 |
| 222528_s_at | SLC25A37 | 2.11E-16 | 5.9049747 |
| 218136_s_at | SLC25A37 | 2.18E-16 | 4.7100764 |
| 211661_x_at | PTAFR | 2.33E-16 | 4.479185 |
| 225598_at | SLC45A4 | 2.51E-16 | 3.6427677 |
| 217977_at | MSRB1 | 2.85E-16 | 5.1410594 |
| 205627_at | CDA | 3.01E-16 | 4.6992775 |
| 207610_s_at | EMR2 | 3.31E-16 | 6.108307 |
| 203042_at | LAMP2 | 3.31E-16 | 4.9047906 |
| 210773_s_at | FPR2 | 3.56E-16 | 4.4555463 |
| 218739_at | ABHD5 | 3.72E-16 | 4.2041522 |
| 211816_x_at | FCAR | 3.72E-16 | 3.3567728 |
| 200919_at | PHC2 | 4.04E-16 | 2.9967746 |
| 211623_s_at | FBL | 4.13E-16 | -5.5416756 |
| 244840_x_at | DOCK4 | 4.4E-16 | 4.170787 |
| 225547_at | SNHG6 | 4.79E-16 | -5.3150672 |
| 232068_s_at | TLR4 | 5.27E-16 | 5.4248523 |
| 210423_s_at | SLC11A1 | 5.97E-16 | 4.8208627 |
| 203041_s_at | LAMP2 | 6E-16 | 5.0495424 |
| 209396_s_at | CHI3L1 | 6.99E-16 | 5.4684285 |
| 202948_at | IL1R1 | 7.41E-16 | 4.4874609 |
| 214465_at | ORM2///ORM1 | 7.44E-16 | 2.8048797 |
| 242335_at | SLC25A37 | 7.46E-16 | 5.7007894 |
| 214141_x_at | SRSF7 | 8.1E-16 | -3.91374 |
| 205922_at | VNN2 | 8.26E-16 | 6.2702877 |
| 208683_at | CAPN2 | 8.93E-16 | -5.9251776 |
| 211037_s_at | MBOAT7 | 1.1E-15 | 3.1018107 |
| 225698_at | EPB41L4A-AS1 | 1.13E-15 | -5.9346394 |
| 223334_at | TMEM126A | 1.13E-15 | -3.5498813 |
| 214042_s_at | RPL22 | 1.21E-15 | -7.174674 |
| 219788_at | PILRA | 1.23E-15 | 5.2480598 |
| 204255_s_at | VDR | 1.28E-15 | 3.4263631 |
| 1553514_a_at | VNN3 | 1.34E-15 | 4.1898404 |
| 200834_s_at | RPS21 | 1.6E-15 | -5.0091863 |
| 236295_s_at | NLRC3 | 1.69E-15 | -5.0349446 |
| 214280_x_at | HNRNPA1 | 1.92E-15 | -5.1386143 |
| 201522_x_at | SNURF///SNRPN | 2.13E-15 | -3.8088513 |
| 210724_at | EMR3 | 2.21E-15 | 4.4078293 |
| 209395_at | CHI3L1 | 2.21E-15 | 6.8145429 |
| 212360_at | AMPD2 | 2.85E-15 | 4.2077473 |
| 221488_s_at | CUTA | 3.24E-15 | -3.2254404 |
| 215078_at | SOD2 | 3.33E-15 | 6.1440774 |
| 201651_s_at | PACSIN2 | 3.41E-15 | 3.8644106 |
| 200029_at | RPL19 | 3.41E-15 | -4.1519607 |
| 222529_at | SLC25A37 | 3.9E-15 | 5.4648644 |
| 220404_at | GPR97 | 3.9E-15 | 4.7931494 |
| 226247_at | PLEKHA1 | 3.97E-15 | -3.6043183 |
| 226275_at | MXD1 | 4.02E-15 | 5.5939374 |
| 225155_at | SNHG5 | 4.04E-15 | -5.1105248 |
| 226131_s_at | RPS16 | 4.04E-15 | -2.6470069 |
| 1553723_at | GPR97 | 4.17E-15 | 3.4964659 |
| 204102_s_at | EEF2 | 4.34E-15 | -4.0559054 |
| 202483_s_at | RANBP1 | 4.42E-15 | -3.9319109 |
| 202988_s_at | RGS1 | 4.73E-15 | -6.5497474 |
| 222686_s_at | CPPED1 | 4.73E-15 | 4.2121383 |
| 209238_at | STX3 | 4.77E-15 | 4.0452191 |
| 201004_at | SSR4 | 5.35E-15 | -3.6318947 |
| 217167_x_at | GK | 5.35E-15 | 3.6059214 |
| 206643_at | HAL | 5.63E-15 | 4.7671461 |
| 223191_at | COX16 | 5.63E-15 | -4.5127535 |
| 218200_s_at | NDUFB2 | 6.38E-15 | -3.9420316 |
| 202990_at | PYGL | 6.51E-15 | 5.3404759 |
| 200651_at | GNB2L1 | 7.47E-15 | -4.9021169 |
| 218237_s_at | SLC38A1 | 7.7E-15 | -4.4514752 |
| 225629_s_at | ZBTB4 | 8.13E-15 | -4.0827298 |
| 215977_x_at | GK | 8.42E-15 | 3.4086674 |
| 223405_at | NPL | 8.76E-15 | 4.4432444 |
| 243296_at | NAMPT | 8.76E-15 | 5.2911143 |
| 211982_x_at | XPO6 | 8.78E-15 | 2.6823968 |
| 226749_at | MRPS9 | 8.92E-15 | -2.7196826 |
| 233085_s_at | NABP1 | 9.84E-15 | 3.7459131 |
| 212860_at | ZDHHC18 | 1.05E-14 | 3.6223312 |
| 204669_s_at | RNF24 | 1.06E-14 | 3.6016278 |
| 216384_x_at | LOC728026///LOC441454///PTMAP5///PTMA | 1.06E-14 | -1.9858161 |
| 1553570_x_at | COX2 | 1.25E-14 | -2.7488306 |
| 228053_s_at | TOMM5 | 1.32E-14 | -4.4094917 |
| 241368_at | PLIN5 | 1.33E-14 | 2.9877321 |
| 212692_s_at | LRBA | 1.33E-14 | -3.1336913 |
| 204007_at | FCGR3B | 1.35E-14 | 8.8238824 |
| 201697_s_at | DNMT1 | 1.35E-14 | -4.4265278 |
| 232629_at | PROK2 | 1.35E-14 | 6.7408136 |
| 202510_s_at | TNFAIP2 | 1.35E-14 | 5.7402434 |
| 34210_at | CD52 | 1.36E-14 | -7.6557056 |
| 220088_at | C5AR1 | 1.44E-14 | 6.3841879 |
| 1555167_s_at | NAMPT | 1.47E-14 | 5.3785022 |
| 1552798_a_at | TLR4 | 1.56E-14 | 3.5443355 |
| 218610_s_at | CPPED1 | 1.57E-14 | 5.3177064 |
| 214866_at | PLAUR | 1.57E-14 | 3.6255247 |
| 212296_at | PSMD14 | 1.57E-14 | -3.1396876 |
| 233587_s_at | SIPA1L2 | 1.6E-14 | 3.5712983 |
| 219748_at | TREML2 | 1.63E-14 | 2.3411171 |
| 205931_s_at | CREB5 | 2.02E-14 | 4.2720223 |
| 214321_at | NOV | 2.31E-14 | 4.1997551 |
| 1553297_a_at | CSF3R | 2.56E-14 | 4.9767069 |
| 202268_s_at | NAE1 | 2.67E-14 | -4.2787853 |
| 224579_at | SLC38A1 | 2.76E-14 | -4.9019666 |
| 206336_at | CXCL6 | 2.94E-14 | 3.5535611 |
| 224833_at | ETS1 | 2.99E-14 | -4.7105928 |
| 204494_s_at | C15orf39 | 3.02E-14 | 3.0228298 |
| 229560_at | TLR8 | 3.37E-14 | 6.7009897 |
| 208646_at | RPS14P3 | 3.37E-14 | -4.3941205 |
| 225373_at | C10orf54 | 3.5E-14 | 3.5748771 |
| 206025_s_at | TNFAIP6 | 3.94E-14 | 7.5142782 |
| 203153_at | IFIT1 | 4.11E-14 | 4.6242692 |
| 239135_at | CPPED1 | 4.23E-14 | 5.6727614 |
| 218201_at | NDUFB2 | 4.33E-14 | -3.5087363 |
| 226179_at | SLC25A37 | 4.43E-14 | 4.8827883 |
| 204661_at | CD52 | 4.55E-14 | -6.8292792 |
| 218403_at | TRIAP1 | 4.62E-14 | -3.6009023 |
| 224707_at | CYSTM1 | 4.69E-14 | 4.5407218 |
| 203167_at | TIMP2 | 4.85E-14 | 3.7488239 |
| 202205_at | VASP | 4.93E-14 | 3.4204738 |
| 201089_at | ATP6V1B2 | 4.93E-14 | 3.3423384 |
| 226817_at | DSC2 | 4.93E-14 | 4.2705481 |
| 218140_x_at | SRPRB | 4.95E-14 | -3.4350757 |
| 1555952_at | SLC19A1 | 5.11E-14 | 2.4687723 |
| 224341_x_at | TLR4 | 5.22E-14 | 6.1662281 |
| 219066_at | PPCDC | 5.27E-14 | 4.0963191 |
| 225414_at | RNF149 | 5.34E-14 | 3.783676 |
| 208887_at | EIF3G | 5.39E-14 | -2.9715271 |
| 226507_at | PAK1 | 5.69E-14 | 3.0148563 |
| 222062_at | IL27RA | 5.7E-14 | -2.934835 |
| 205844_at | VNN1 | 5.86E-14 | 4.4867428 |
| 224815_at | COMMD7 | 5.9E-14 | -2.8436009 |
| 227184_at | PTAFR | 5.9E-14 | 5.0257246 |
| 200965_s_at | ABLIM1 | 5.96E-14 | -4.6516988 |
| 206209_s_at | CA4 | 5.96E-14 | 3.3863025 |
| 201272_at | AKR1B1 | 6.32E-14 | -2.6021011 |
| 237591_at | LINC00173 | 6.35E-14 | 3.904585 |
| 208688_x_at | EIF3B | 6.5E-14 | -3.1205661 |
| 1553569_at | COX2 | 6.62E-14 | -2.7975818 |
| 218660_at | DYSF | 6.83E-14 | 3.8591149 |
| 211986_at | AHNAK | 7.06E-14 | -3.2642956 |
| 228949_at | WLS | 7.06E-14 | 4.1681614 |
| 218435_at | DNAJC15 | 7.54E-14 | -3.9117574 |
| 212506_at | PICALM | 7.91E-14 | 3.1367184 |
| 221920_s_at | SLC25A37 | 8.24E-14 | 6.1312263 |
| 227250_at | KREMEN1 | 8.72E-14 | 3.0923364 |
| 223329_x_at | SUGT1 | 8.73E-14 | -3.3135471 |
| 212846_at | RRP1B | 8.75E-14 | -4.0869945 |
| 227438_at | ALPK1 | 9.13E-14 | 2.5801837 |
| 226489_at | TMCC3 | 9.13E-14 | 4.2673892 |
| 224309_s_at | SUGT1 | 9.13E-14 | -3.4175878 |
| 224825_at | DNTTIP1 | 9.19E-14 | 3.2920074 |
| 219434_at | TREM1 | 9.69E-14 | 7.4397287 |
| 212922_s_at | SMYD2 | 1E-13 | -3.2852901 |
| 206026_s_at | TNFAIP6 | 1.03E-13 | 7.9155564 |
| 218978_s_at | SLC25A37 | 1.07E-13 | 2.9304382 |
| 58780_s_at | ARHGEF40 | 1.08E-13 | 4.2481259 |
| 212657_s_at | IL1RN | 1.08E-13 | 8.4282382 |
| 205590_at | RASGRP1 | 1.1E-13 | -6.1311965 |
| 210201_x_at | BIN1 | 1.1E-13 | -2.612233 |
| 209795_at | CD69 | 1.15E-13 | -6.2568126 |
| 205003_at | DOCK4 | 1.17E-13 | 3.0666248 |
| 221011_s_at | LBH | 1.18E-13 | -4.5223206 |
| 244578_at | LCP2 | 1.24E-13 | 3.3410414 |
| 205174_s_at | QPCT | 1.27E-13 | 6.2373125 |
| 200010_at | RPL11 | 1.32E-13 | -3.8690917 |
| 201365_at | OAZ2 | 1.33E-13 | 3.1782779 |
| 202546_at | VAMP8 | 1.34E-13 | -4.6641648 |
| 223207_x_at | PHPT1 | 1.39E-13 | -2.9076485 |
| 235514_at | ASPRV1 | 1.41E-13 | 2.5776611 |
| 224719_s_at | C12orf57 | 1.41E-13 | -6.1222787 |
| 202084_s_at | SEC14L1 | 1.41E-13 | 4.2402117 |
| 207008_at | CXCR2 | 1.46E-13 | 8.2175408 |
| 222218_s_at | PILRA | 1.47E-13 | 5.5628243 |
| 219279_at | DOCK10 | 1.47E-13 | -4.0429544 |
| 209313_at | GPN1 | 1.52E-13 | -2.1465789 |
| 209288_s_at | CDC42EP3 | 1.64E-13 | 3.8491615 |
| 223852_s_at | STK40 | 1.67E-13 | 3.4780636 |
| 213947_s_at | NUP210 | 1.67E-13 | -2.6493971 |
| 222662_at | PPP1R3B | 1.75E-13 | 3.2808429 |
| 220302_at | MAK | 1.75E-13 | 3.4103566 |
| 207085_x_at | CSF2RA | 1.77E-13 | 3.324507 |
| 214746_s_at | ZNF467 | 1.89E-13 | 3.4136656 |
| 207507_s_at | ATP5G3 | 1.95E-13 | -3.9148635 |
| 208635_x_at | NACA | 2.24E-13 | -2.5088165 |
| 205568_at | AQP9 | 2.24E-13 | 7.271643 |
| 212646_at | RFTN1 | 2.25E-13 | -4.1871734 |
| 223944_at | NLRP12 | 2.26E-13 | 2.7663802 |
| 200735_x_at | NACA | 2.35E-13 | -2.4981913 |
| 207857_at | LILRA2 | 2.37E-13 | 6.1659756 |
| 200675_at | CD81 | 2.42E-13 | -5.3422702 |
| 203433_at | MTHFS | 2.53E-13 | 2.8858183 |
| 207643_s_at | TNFRSF1A | 2.54E-13 | 4.5477392 |
| 218950_at | ARAP3 | 2.57E-13 | 3.5052363 |
| 208663_s_at | TTC3 | 2.63E-13 | -3.3101466 |
| 207094_at | CXCR1 | 2.69E-13 | 2.8955622 |
| 202897_at | SIRPA | 2.7E-13 | 4.3605239 |
| 207275_s_at | ACSL1 | 2.72E-13 | 6.1122505 |
| 228490_at | ABHD2 | 2.72E-13 | 3.8729 |
| 205119_s_at | FPR1 | 2.87E-13 | 7.2171495 |
| 208833_s_at | ATXN10 | 2.87E-13 | -3.0523792 |
| 213935_at | ABHD5 | 2.88E-13 | 3.5776851 |
| 220421_at | BTNL8 | 2.93E-13 | 3.4052037 |
| 217870_s_at | CMPK1 | 2.97E-13 | -3.2896573 |
| 214784_x_at | XPO6 | 3.12E-13 | 2.3660434 |
| 204174_at | ALOX5AP | 3.14E-13 | 3.9563355 |
| 206059_at | ZNF91 | 3.14E-13 | -4.5706407 |
| 210789_x_at | CEACAM3 | 3.33E-13 | 2.2826466 |
| 234926_s_at | RTFDC1 | 3.39E-13 | 2.1527834 |
| 213607_x_at | NADK | 3.46E-13 | 2.816435 |
| 226671_at | LAMP2 | 3.62E-13 | 3.7056623 |
| 230425_at | EPHB1 | 3.69E-13 | 3.0885386 |
| 222297_x_at | RPL18 | 3.79E-13 | -4.9416039 |
| 218035_s_at | RBM47 | 3.87E-13 | 5.364002 |
| 224327_s_at | DGAT2 | 4E-13 | 3.2459965 |
| 204493_at | BID | 4.12E-13 | 4.9532347 |
| 222872_x_at | NABP1 | 4.19E-13 | 3.4374948 |
| 211100_x_at | LILRA2 | 4.19E-13 | 3.4389259 |
| 210564_x_at | CFLAR | 4.2E-13 | 3.0261801 |
| 221210_s_at | NPL | 4.24E-13 | 4.228432 |
| 214439_x_at | BIN1 | 4.4E-13 | -2.7943822 |
| 202771_at | PIEZO1 | 4.48E-13 | -3.0235489 |
| 204744_s_at | IARS | 4.52E-13 | -4.3366353 |
| 204254_s_at | VDR | 4.55E-13 | 3.9343938 |
| 217912_at | DUS1L | 4.77E-13 | -2.6238494 |
| 219183_s_at | CYTH4 | 4.8E-13 | 2.7022742 |
| 201947_s_at | CCT2 | 4.81E-13 | -3.2869829 |
| 204839_at | POP5 | 5E-13 | -3.7109411 |
| 202856_s_at | SLC16A3 | 5E-13 | 4.234555 |
| 212252_at | CAMKK2 | 5.08E-13 | 2.2686218 |
| 219162_s_at | MRPL11 | 5.16E-13 | -2.3276539 |
| 225325_at | MFSD6 | 5.25E-13 | -3.5188994 |
| 220832_at | TLR8 | 5.26E-13 | 3.5160093 |
| 207765_s_at | FAM214B | 5.3E-13 | 2.1515182 |
| 36564_at | RNF19B | 5.3E-13 | 3.5927718 |
| 206222_at | TNFRSF10C | 5.31E-13 | 3.481046 |
| 210706_s_at | RNF24 | 5.36E-13 | 2.6085845 |
| 229164_s_at | ABTB1 | 5.46E-13 | 2.67538 |
| 212750_at | PPP1R16B | 5.48E-13 | -4.3989068 |
| 218005_at | ZNF22 | 5.68E-13 | -3.7859657 |
| 205227_at | IL1RAP | 5.93E-13 | 2.9241359 |
| 225056_at | SIPA1L2 | 5.99E-13 | 3.9967789 |
| 203542_s_at | KLF9 | 6.45E-13 | -3.7581181 |
| 200064_at | HSP90AB1 | 6.72E-13 | -3.9091044 |
| 202931_x_at | BIN1 | 7.22E-13 | -2.6195644 |
| 205896_at | SLC22A4 | 7.22E-13 | 4.6638756 |
| 201138_s_at | SSB | 7.22E-13 | -3.3834936 |
| 209498_at | CEACAM1 | 7.27E-13 | 3.0963335 |
| 213649_at | SRSF7 | 7.27E-13 | -4.1234824 |
| 220486_x_at | TMEM164 | 7.37E-13 | 2.0812744 |
| 203484_at | SEC61G | 7.39E-13 | -2.8092869 |
| 219024_at | PLEKHA1 | 7.57E-13 | -3.0068384 |
| 202974_at | MPP1 | 7.68E-13 | 2.954858 |
| 201364_s_at | OAZ2 | 7.76E-13 | 3.5510847 |
| 213038_at | RNF19B | 7.81E-13 | 3.9808767 |
| 55705_at | R3HDM4 | 8.13E-13 | 1.5677448 |
| 228758_at | BCL6 | 8.33E-13 | 3.9268392 |
| 202833_s_at | SERPINA1 | 8.58E-13 | 6.5792995 |
| 217731_s_at | ITM2B | 8.94E-13 | 2.2704868 |
| 241809_at | FAM212B | 9.13E-13 | 3.5903997 |
| 206420_at | IGSF6 | 1.03E-12 | 6.6954498 |
| 203853_s_at | GAB2 | 1.07E-12 | 3.381306 |
| 200042_at | RTCB | 1.1E-12 | -2.2848078 |
| 208308_s_at | GPI | 1.19E-12 | -3.1053573 |
| 212831_at | MEGF9 | 1.21E-12 | 3.3918745 |
| 202741_at | PRKACB | 1.22E-12 | -4.4322315 |
| 221060_s_at | TLR4 | 1.22E-12 | 3.9475627 |
| 226024_at | COMMD1 | 1.22E-12 | -2.6025403 |
| 222934_s_at | CLEC4E | 1.24E-12 | 5.8405761 |
| 225612_s_at | B3GNT5 | 1.32E-12 | 5.9264587 |
| 205479_s_at | PLAU | 1.34E-12 | 5.5067458 |
| 211317_s_at | CFLAR | 1.39E-12 | 3.2969029 |
| 220001_at | PADI4 | 1.4E-12 | 4.5514451 |
| 1552772_at | CLEC4D | 1.46E-12 | 3.2473071 |
| 213982_s_at | RABGAP1L | 1.55E-12 | -2.8035185 |
| 203702_s_at | TTLL4 | 1.56E-12 | 1.9656704 |
| 201853_s_at | CDC25B | 1.59E-12 | -3.1212207 |
| 202737_s_at | LSM4 | 1.65E-12 | -2.6454256 |
| 208485_x_at | CFLAR | 1.68E-12 | 2.9340411 |
| 211561_x_at | MAPK14 | 1.71E-12 | 2.2154957 |
| 214017_s_at | DHX34 | 1.77E-12 | 2.6373072 |
| 222040_at | HNRNPA1 | 1.79E-12 | -4.7609787 |
| 204351_at | S100P | 2.04E-12 | 7.8204189 |
| 225597_at | SLC45A4 | 2.08E-12 | 4.7883468 |
| 227228_s_at | CCDC88C | 2.08E-12 | -2.9208632 |
| 217737_x_at | RTFDC1 | 2.1E-12 | 1.981823 |
| 200642_at | SOD1 | 2.11E-12 | -4.0943364 |
| 219762_s_at | RPL36 | 2.11E-12 | -5.7514555 |
| 206877_at | MXD1 | 2.19E-12 | 5.0922974 |
| 227143_s_at | BID | 2.22E-12 | 4.0329386 |
| 217732_s_at | ITM2B | 2.23E-12 | 2.6778548 |
| 41577_at | PPP1R16B | 2.24E-12 | -4.5558985 |
| 211101_x_at | LILRA2 | 2.27E-12 | 3.4033554 |
| 231908_at | ZDHHC18 | 2.3E-12 | 2.0918779 |
| 211429_s_at | SERPINA1 | 2.3E-12 | 6.1280986 |
| 201362_at | IVNS1ABP | 2.32E-12 | 3.2789106 |
| 201300_s_at | PRNP | 2.33E-12 | -4.3540459 |
| 211862_x_at | CFLAR | 2.39E-12 | 3.0549878 |
| 206208_at | CA4 | 2.39E-12 | 2.6422721 |
| 200094_s_at | EEF2 | 2.39E-12 | -3.8276758 |
| 200872_at | S100A10 | 2.44E-12 | -4.8075125 |
| 220890_s_at | DDX47 | 2.46E-12 | -4.788027 |
| 204949_at | ICAM3 | 2.47E-12 | 2.7129235 |
| 244556_at | LCP2 | 2.48E-12 | 3.485586 |
| 39729_at | PRDX2 | 2.48E-12 | -4.7373335 |
| 200936_at | RPL8 | 2.48E-12 | -3.9791902 |
| 200821_at | LAMP2 | 2.59E-12 | 3.4135893 |
| 217962_at | NOP10 | 2.69E-12 | 3.1228954 |
| 203063_at | PPM1F | 2.84E-12 | 2.2094265 |
| 212276_at | LPIN1 | 2.93E-12 | -3.3563519 |
| 234942_s_at | DNTTIP1 | 2.93E-12 | 3.9336599 |
| 203887_s_at | THBD | 3.07E-12 | 3.6381306 |
| 233842_x_at | RTFDC1 | 3.11E-12 | 2.0308241 |
| 215719_x_at | FAS | 3.27E-12 | 4.1035316 |
| 209038_s_at | EHD1 | 3.35E-12 | 2.4570115 |
| 208749_x_at | FLOT1 | 3.38E-12 | 2.3111002 |
| 201379_s_at | TPD52L2 | 3.42E-12 | 2.811206 |
| 211971_s_at | LRPPRC | 3.42E-12 | -4.0067565 |
| 217833_at | SYNCRIP | 3.42E-12 | -2.7614403 |
| 210190_at | STX11 | 3.44E-12 | 3.2709799 |
| 240440_at | NPL | 3.44E-12 | 3.582182 |
| 223294_at | PBDC1 | 3.44E-12 | -3.9491466 |
| 32541_at | PPP3CC | 3.44E-12 | -3.8513321 |
| 233555_s_at | SULF2 | 3.46E-12 | 3.5655695 |
| 206440_at | LIN7A | 3.46E-12 | 3.1017709 |
| 221895_at | MOSPD2 | 3.56E-12 | 2.6876249 |
| 1556072_at | LINC00528 | 3.65E-12 | 3.814764 |
| 209171_at | ITPA | 3.65E-12 | -2.234531 |
| 223482_at | TMEM120A | 3.76E-12 | 2.4770903 |
| 208304_at | CCR3 | 3.95E-12 | 3.4571734 |
| 210142_x_at | FLOT1 | 4.02E-12 | 2.4693219 |
| 217916_s_at | FAM49B | 4.08E-12 | 2.770211 |
| 203585_at | ZNF185 | 4.09E-12 | 2.659603 |
| 223299_at | SEC11C | 4.1E-12 | -2.2315449 |
| 223454_at | CXCL16 | 4.1E-12 | 3.7606898 |
| 205220_at | HCAR3 | 4.1E-12 | 7.1765996 |
| 201024_x_at | EIF5B | 4.1E-12 | -3.2117126 |
| 200912_s_at | EIF4A2 | 4.12E-12 | -1.887065 |
| 211102_s_at | LILRA2 | 4.12E-12 | 2.5804691 |
| 225310_at | RBMX | 4.21E-12 | -4.2608163 |
| 208670_s_at | EID1 | 4.31E-12 | -3.2046887 |
| 208517_x_at | BTF3 | 4.36E-12 | -2.8844458 |
| 201672_s_at | USP14 | 4.36E-12 | -3.0655406 |
| 217759_at | TRIM44 | 4.48E-12 | -2.9873075 |
| 223552_at | LRRC4 | 4.49E-12 | 2.862736 |
| 211702_s_at | USP32 | 4.59E-12 | 2.4072903 |
| 239598_s_at | LPCAT2 | 4.69E-12 | 2.7053489 |
| 224560_at | TIMP2 | 4.7E-12 | 3.9151376 |
| 210449_x_at | MAPK14 | 4.71E-12 | 2.2115787 |
| 202396_at | TCERG1 | 4.81E-12 | -5.5716122 |
| 212770_at | TLE3 | 4.87E-12 | 2.6552879 |
| 203126_at | IMPA2 | 5.04E-12 | 4.2359906 |
| 1555214_a_at | CLEC7A | 5.08E-12 | 2.8683521 |
| 1554414_a_at | OSGIN2 | 5.08E-12 | 2.1007119 |
| 204072_s_at | FRY | 5.14E-12 | 3.396573 |
| 207508_at | ATP5G3 | 5.27E-12 | -3.741098 |
| 207000_s_at | PPP3CC | 5.29E-12 | -3.0959789 |
| 202101_s_at | RALB | 5.41E-12 | 2.7893363 |
| 209303_at | NDUFS4 | 5.41E-12 | -3.5377642 |
| 219014_at | PLAC8 | 5.44E-12 | -4.451597 |
| 202426_s_at | RXRA | 5.48E-12 | 2.5994993 |
| 226620_x_at | DAZAP1 | 5.49E-12 | -2.6487212 |
| 221698_s_at | CLEC7A | 5.49E-12 | 6.1307671 |
| 211287_x_at | CSF2RA | 5.52E-12 | 2.3397872 |
| 209861_s_at | METAP2 | 5.83E-12 | -3.9730467 |
| 1564637_a_at | FAM98B | 5.87E-12 | -2.7904158 |
| 208783_s_at | CD46 | 5.87E-12 | 2.8718289 |
| 211744_s_at | CD58 | 6.06E-12 | 3.6576143 |
| 211305_x_at | FCAR | 6.11E-12 | 3.2106113 |
| 224008_s_at | KCNK7 | 6.11E-12 | 2.2489396 |
| 211939_x_at | BTF3 | 6.11E-12 | -3.2276963 |
| 200998_s_at | CKAP4 | 6.17E-12 | 3.5944209 |
| 218831_s_at | FCGRT | 6.36E-12 | 3.1752527 |
| 243099_at | NFAM1 | 6.36E-12 | 2.6810619 |
| 1559502_s_at | LRRC25 | 6.36E-12 | 2.4719903 |
| 219460_s_at | TMEM127 | 6.37E-12 | 1.8750823 |
| 202207_at | ARL4C | 6.46E-12 | -7.7532205 |
| 222833_at | LPCAT2 | 6.5E-12 | 3.4318172 |
| 204089_x_at | MAP3K4 | 6.72E-12 | -3.1781953 |
| 211733_x_at | SCP2 | 6.82E-12 | -3.3091006 |
| 219921_s_at | DOCK5 | 6.85E-12 | 2.4440752 |
| 222721_at | CNIH4 | 6.85E-12 | 3.8365092 |
| 206470_at | PLXNC1 | 6.85E-12 | 2.7382636 |
| 216841_s_at | SOD2 | 7.1E-12 | 6.0878258 |
| 221847_at | LOC100129361 | 7.57E-12 | -3.4731158 |
| 226757_at | IFIT2 | 7.75E-12 | 5.4308363 |
| 236172_at | LTB4R | 7.75E-12 | 3.0617878 |
| 217865_at | RNF130 | 7.77E-12 | 3.8020269 |
| 205771_s_at | AKAP7 | 7.88E-12 | -2.2381379 |
| 225220_at | SNHG8 | 8.11E-12 | -4.980597 |
| 224846_at | SHKBP1 | 8.18E-12 | 2.1655181 |
| 224948_at | MRPS24 | 8.18E-12 | -3.2340529 |
| 211725_s_at | BID | 8.37E-12 | 4.6085578 |
| 226726_at | MBOAT2 | 8.37E-12 | 3.0889194 |
| 33323_r_at | SFN | 8.44E-12 | 2.6594802 |
| 234644_x_at | TNFRSF10C | 8.53E-12 | 3.008619 |
| 223154_at | MRPL1 | 8.7E-12 | -3.3374585 |
| 203044_at | CHSY1 | 8.7E-12 | 3.5065678 |
| 213399_x_at | RPN2 | 8.7E-12 | -2.6239943 |
| 1554997_a_at | PTGS2 | 8.75E-12 | 6.612186 |
| 201189_s_at | ITPR3 | 8.82E-12 | -2.3407625 |
| 209600_s_at | ACOX1 | 8.93E-12 | 2.9847858 |
| 206522_at | MGAM | 9.1E-12 | 6.8409419 |
| 225631_at | EEPD1 | 9.87E-12 | 1.8527977 |
| 209286_at | CDC42EP3 | 1E-11 | 4.0226672 |
| 1554691_a_at | PACSIN2 | 1E-11 | 1.7775356 |
| 224412_s_at | TRPM6 | 1.03E-11 | 2.618903 |
| 1555851_s_at | SEPW1 | 1.03E-11 | -2.7490693 |
| 216252_x_at | FAS | 1.03E-11 | 3.6394335 |
| 203774_at | MTR | 1.05E-11 | -4.4200699 |
| 209508_x_at | CFLAR | 1.06E-11 | 2.9076413 |
| 226248_s_at | KIAA1324 | 1.08E-11 | 3.8140115 |
| 203194_s_at | NUP98 | 1.1E-11 | 2.3165477 |
| 211316_x_at | CFLAR | 1.15E-11 | 2.9852965 |
| 216942_s_at | CD58 | 1.16E-11 | 3.8307802 |
| 204546_at | KIAA0513 | 1.19E-11 | 2.7627174 |
| 221958_s_at | WLS | 1.2E-11 | 3.9742428 |
| 212274_at | LPIN1 | 1.21E-11 | -3.1910189 |
| 213716_s_at | SECTM1 | 1.22E-11 | 3.3852682 |
| 223015_at | EIF2A | 1.27E-11 | -2.8325819 |
| 205312_at | SPI1 | 1.29E-11 | 2.5147285 |
| 225372_at | C10orf54 | 1.29E-11 | 4.1440841 |
| 220755_s_at | C6orf48 | 1.29E-11 | -4.2231096 |
| 218398_at | MRPS30 | 1.29E-11 | -3.8609093 |
| 212511_at | PICALM | 1.29E-11 | 3.3224662 |
| 218902_at | NOTCH1 | 1.3E-11 | 2.8014832 |
| 201909_at | RPS4Y1 | 1.31E-11 | -4.0437987 |
| 225001_at | RAB3D | 1.31E-11 | 2.9110314 |
| 222405_at | PTPLAD1 | 1.32E-11 | -2.323907 |
| 202754_at | R3HDM1 | 1.32E-11 | -2.2265729 |
| 219594_at | NINJ2 | 1.35E-11 | 3.2402966 |
| 229813_x_at | DAZAP1 | 1.35E-11 | -2.7691152 |
| 201837_s_at | SUPT7L | 1.4E-11 | -2.6243481 |
| 221477_s_at | SOD2 | 1.41E-11 | 4.6951034 |
| 201812_s_at | C4orf46///TOMM7 | 1.41E-11 | -3.5614635 |
| 213812_s_at | CAMKK2 | 1.41E-11 | 1.6200054 |
| 212826_s_at | SLC25A6 | 1.41E-11 | -4.1407978 |
| 230100_x_at | PAK1 | 1.43E-11 | 2.2989336 |
| 203021_at | SLPI | 1.45E-11 | 3.9764918 |
| 222550_at | ARMC1 | 1.46E-11 | -2.9633394 |
| 208981_at | PECAM1 | 1.46E-11 | 3.7529192 |
| 201068_s_at | PSMC2 | 1.51E-11 | -2.9045669 |
| 205324_s_at | FTSJ1 | 1.51E-11 | -2.9127328 |
| 206393_at | TNNI2 | 1.53E-11 | 3.0833531 |
| 202136_at | ZMYND11 | 1.53E-11 | -2.6434113 |
| 223162_s_at | KIAA1147 | 1.53E-11 | -3.1752086 |
| 208787_at | MRPL3 | 1.53E-11 | -3.9756702 |
| 209467_s_at | MKNK1 | 1.56E-11 | 1.7734388 |
| 217956_s_at | ENOPH1 | 1.57E-11 | -3.6894979 |
| 211286_x_at | CSF2RA | 1.57E-11 | 2.482394 |
| 226577_at | PSEN1 | 1.57E-11 | 1.7682578 |
| 203462_x_at | EIF3B | 1.6E-11 | -2.419553 |
| 226811_at | FAM46C | 1.61E-11 | -4.679241 |
| 200978_at | MDH1 | 1.64E-11 | -2.70616 |
| 224055_x_at | KCNK7 | 1.71E-11 | 1.888513 |
| 209930_s_at | NFE2 | 1.75E-11 | 3.9172444 |
| 213794_s_at | NGDN | 1.79E-11 | -3.2035131 |
| 203436_at | RPP30 | 1.79E-11 | -1.6703672 |
| 212114_at | ATXN7L3B | 1.83E-11 | -2.5890973 |
| 222431_at | SPIN1 | 1.87E-11 | -2.7759675 |
| 201328_at | ETS2 | 1.89E-11 | 3.9333422 |
| 243699_at | LOC100507006 | 1.9E-11 | 2.778839 |
| 213817_at | IRAK3 | 1.93E-11 | 3.7386402 |
| 206115_at | EGR3 | 1.94E-11 | 3.8742204 |
| 219161_s_at | CKLF | 1.94E-11 | 2.7250602 |
| 201315_x_at | IFITM2 | 1.96E-11 | 4.209665 |
| 216862_s_at | CMC4 | 1.99E-11 | -2.3071968 |
| 212830_at | MEGF9 | 2.01E-11 | 4.50254 |
| 202475_at | TMEM147 | 2.1E-11 | -2.5677719 |
| 239213_at | SERPINB1 | 2.13E-11 | 2.3126986 |
| 200052_s_at | ILF2 | 2.19E-11 | -3.8589023 |
| 213805_at | ABHD5 | 2.28E-11 | 3.6908435 |
| 210027_s_at | APEX1 | 2.3E-11 | -4.1683936 |
| 227948_at | FGD4 | 2.32E-11 | 3.3459286 |
| 224903_at | CIRH1A | 2.4E-11 | -3.9298844 |
| 226243_at | PTRHD1 | 2.4E-11 | -3.3010844 |
| 1569149_at | PDLIM7 | 2.42E-11 | 3.1765504 |
| 210633_x_at | KRT10 | 2.42E-11 | -2.9451312 |
| 209682_at | CBLB | 2.43E-11 | -3.3774218 |
| 222496_s_at | RBM47 | 2.43E-11 | 3.8308995 |
| 201569_s_at | SAMM50 | 2.47E-11 | -2.4931791 |
| 208918_s_at | NADK | 2.48E-11 | 4.432499 |
| 220179_at | DPEP3 | 2.52E-11 | 2.2898142 |
| 210452_x_at | CYP4F2 | 2.57E-11 | 2.0641486 |
| 225967_s_at | C17orf89 | 2.6E-11 | -2.6218491 |
| 223502_s_at | TNFSF13B | 2.64E-11 | 5.3598442 |
| 205645_at | REPS2 | 2.65E-11 | 3.0124386 |
| 225398_at | RPUSD4 | 2.65E-11 | -2.3028456 |
| 1555953_at | SLC19A1 | 2.65E-11 | 2.03378 |
| 205707_at | IL17RA | 2.68E-11 | 1.9910628 |
| 228059_x_at | MRPS22 | 2.7E-11 | -2.4620923 |
| 207205_at | CEACAM4 | 2.71E-11 | 1.9110339 |
| 210904_s_at | IL13RA1 | 2.73E-11 | 3.7989573 |
| 222266_at | URI1 | 2.74E-11 | -3.3355019 |
| 218132_s_at | TSEN34 | 2.84E-11 | 3.0928045 |
| 209181_s_at | RABGGTB | 2.85E-11 | -4.5592063 |
| 213491_x_at | RPN2 | 2.85E-11 | -2.3396608 |
| 209034_at | PNRC1 | 2.85E-11 | 1.8383127 |
| 204542_at | ST6GALNAC2 | 2.87E-11 | 2.7852017 |
| 225017_at | CCDC14 | 2.95E-11 | -2.5999793 |
| 225768_at | NR1D2 | 2.97E-11 | -4.7045168 |
| 208018_s_at | HCK | 2.98E-11 | 5.6957531 |
| 228332_s_at | C11orf31 | 3.02E-11 | -4.5148751 |
| 203259_s_at | HDDC2 | 3.02E-11 | -3.3857086 |
| 209486_at | UTP3 | 3.07E-11 | -3.7316445 |
| 220740_s_at | SLC12A6 | 3.11E-11 | 3.5168139 |
| 204061_at | PRKX | 3.11E-11 | -3.5139838 |
| 230860_at | CEP19 | 3.12E-11 | 3.6938116 |
| 214173_x_at | URI1 | 3.15E-11 | -2.8161758 |
| 240572_s_at | LOC374443 | 3.21E-11 | -3.9937774 |
| 224876_at | C5orf24 | 3.22E-11 | -1.8258239 |
| 220187_at | STEAP4 | 3.23E-11 | 3.7793287 |
| 213846_at | COX7C | 3.24E-11 | -4.7741798 |
| 220933_s_at | ZCCHC6 | 3.31E-11 | 2.8112521 |
| 216199_s_at | MAP3K4 | 3.31E-11 | -4.0777069 |
| 225029_at | LINC01420 | 3.32E-11 | -3.2740868 |
| 212414_s_at | GLYR1///SEPT6 | 3.35E-11 | -3.2258904 |
| 210242_x_at | ST20 | 3.35E-11 | 1.5940208 |
| 216570_x_at | RPL29 | 3.36E-11 | -3.3226684 |
| 225153_at | GFM1 | 3.36E-11 | -2.6679896 |
| 207628_s_at | WBSCR22 | 3.37E-11 | -2.7317988 |
| 235568_at | MCEMP1 | 3.4E-11 | 3.4017383 |
| 243954_at | LINC00877 | 3.47E-11 | 2.1355286 |
| 212516_at | ARAP1 | 3.51E-11 | 2.6446514 |
| 220066_at | NOD2 | 3.58E-11 | 3.5288408 |
| 206934_at | SIRPB1 | 3.6E-11 | 3.0990548 |
| 211783_s_at | MTA1 | 3.61E-11 | -3.3600883 |
| 224765_at | MSL1 | 3.65E-11 | 3.2798932 |
| 201668_x_at | MARCKS | 3.72E-11 | 2.6037596 |
| 213892_s_at | APRT | 3.81E-11 | -2.7798007 |
| 218456_at | CAPRIN2 | 3.87E-11 | -1.9779473 |
| 200914_x_at | KTN1 | 3.88E-11 | -3.0315393 |
| 209882_at | RIT1 | 3.96E-11 | 3.4316984 |
| 202197_at | MTMR3 | 3.99E-11 | 1.6536702 |
| 222493_s_at | ZFAND3 | 4.01E-11 | 1.7868418 |
| 216336_x_at | LOC100505584///MT1E | 4.12E-11 | 3.6104011 |
| 213524_s_at | G0S2 | 4.21E-11 | 7.9974439 |
| 208801_at | SRP72 | 4.37E-11 | -3.1461887 |
| 1570078_a_at | DOCK5 | 4.37E-11 | 2.4142782 |
| 203049_s_at | TTC37 | 4.38E-11 | -4.0573238 |
| 210629_x_at | LST1 | 4.41E-11 | 5.6138532 |
| 202241_at | TRIB1 | 4.46E-11 | 6.3700908 |
| 231861_at | LRP10 | 4.49E-11 | 2.7347298 |
| 227889_at | LPCAT2 | 4.59E-11 | 3.7546327 |
| 205118_at | FPR1 | 4.61E-11 | 3.2667606 |
| 201339_s_at | SCP2 | 4.62E-11 | -3.1859926 |
| 202545_at | PRKCD | 4.64E-11 | 3.1413022 |
| 1552828_at | SNX18 | 4.67E-11 | 1.6356352 |
| 238439_at | ANKRD22 | 4.75E-11 | 2.6795146 |
| 218230_at | ARFIP1 | 5.09E-11 | 3.299549 |
| 204690_at | STX8 | 5.14E-11 | -2.3711701 |
| 208982_at | PECAM1 | 5.21E-11 | 3.7387854 |
| 209143_s_at | CLNS1A | 5.25E-11 | -4.1630155 |
| 218307_at | RSAD1 | 5.28E-11 | -2.4484472 |
| 201590_x_at | ANXA2 | 5.32E-11 | -4.0235858 |
| 226364_at | HIP1 | 5.44E-11 | 2.8546025 |
| 208466_at | RAB3D | 5.44E-11 | 1.6344902 |
| 214084_x_at | NCF1C | 5.44E-11 | 4.7611067 |
| 221970_s_at | NOL11 | 5.46E-11 | -3.1409861 |
| 212441_at | KIAA0232 | 5.75E-11 | 2.500002 |
| 206050_s_at | RNH1 | 5.76E-11 | -1.4639941 |
| 209615_s_at | PAK1 | 5.87E-11 | 3.3634736 |
| 225556_at | VMA21 | 5.98E-11 | -3.7547262 |
| 217964_at | TTC19 | 5.98E-11 | -3.1940606 |
| 223303_at | FERMT3 | 6.06E-11 | 1.960275 |
| 202542_s_at | AIMP1 | 6.25E-11 | -3.5081292 |
| 201900_s_at | AKR1A1 | 6.37E-11 | -2.5605323 |
| 217846_at | QARS | 6.53E-11 | -3.3452063 |
| 204951_at | RHOH | 6.65E-11 | -3.8054945 |
| 201993_x_at | HNRNPDL | 6.65E-11 | -2.3132146 |
| 1560527_at | NFE4 | 6.82E-11 | 3.0839921 |
| 37384_at | PPM1F | 6.85E-11 | 2.2206587 |
| 214109_at | LRBA | 6.93E-11 | -2.0941307 |
| 226872_at | RFX2 | 6.97E-11 | 2.8429314 |
| 212606_at | WDFY3 | 7.04E-11 | 3.9067813 |
| 223006_s_at | TMEM245 | 7.08E-11 | -3.4597786 |
| 1559777_at | LOC731424 | 7.12E-11 | 2.8645936 |
| 1555643_s_at | LILRA5 | 7.34E-11 | 4.0176971 |
| 224366_s_at | REPS1 | 7.34E-11 | -2.3121625 |
| 218366_x_at | METTL17 | 7.37E-11 | -1.9852693 |
| 221058_s_at | CKLF | 7.48E-11 | 2.7235952 |
| 224677_x_at | C11orf31 | 7.55E-11 | -3.595576 |
| 1563071_at | LOC100289061 | 7.65E-11 | 1.6872247 |
| 201458_s_at | BUB3 | 7.74E-11 | -2.9016705 |
| 202855_s_at | SLC16A3 | 7.75E-11 | 2.3444632 |
| 209949_at | NCF2 | 7.76E-11 | 6.7598522 |
| 205566_at | ABHD2 | 7.92E-11 | 2.5146727 |
| 208986_at | TCF12 | 7.94E-11 | -2.131307 |
| 201179_s_at | GNAI3 | 7.98E-11 | 2.5104941 |
| 203814_s_at | NQO2 | 7.98E-11 | 3.4466576 |
| 228408_s_at | SDAD1 | 8.02E-11 | -4.3384559 |
| 221039_s_at | ASAP1 | 8.08E-11 | 2.4534521 |
| 231045_x_at | C11orf31 | 8.13E-11 | -3.3925655 |
| 204436_at | PLEKHO2 | 8.15E-11 | 2.3834533 |
| 218084_x_at | FXYD5 | 8.31E-11 | -2.6135931 |
| 223451_s_at | CKLF | 8.31E-11 | 3.3085853 |
| 234339_s_at | GLTSCR2 | 8.32E-11 | -3.5571675 |
| 215838_at | LILRA5 | 8.37E-11 | 4.1864894 |
| 221484_at | B4GALT5 | 8.37E-11 | 2.9551176 |
| 201750_s_at | ECE1 | 8.38E-11 | 1.4044585 |
| 224364_s_at | PPIL3 | 8.39E-11 | -3.0671468 |
| 218566_s_at | CHORDC1 | 8.46E-11 | -2.976736 |
| 224628_at | ERLEC1 | 8.6E-11 | -2.1123869 |
| 212248_at | MTDH | 8.78E-11 | -3.2861388 |
| 226673_at | SH2D3C | 8.8E-11 | 2.5795158 |
| 201412_at | LRP10 | 8.85E-11 | 2.5791609 |
| 224724_at | SULF2 | 8.88E-11 | 4.5906592 |
| 212229_s_at | FBXO21 | 8.94E-11 | -3.3757779 |
| 225534_at | SMIM19 | 9.17E-11 | -2.4868549 |
| 41220_at | 42256 | 9.32E-11 | -1.7678838 |
| 227930_at | AGO4 | 9.33E-11 | 2.6954484 |
| 223000_s_at | F11R | 9.34E-11 | 2.4770245 |
| 203200_s_at | MTRR | 9.42E-11 | -3.2009948 |
| 226845_s_at | MYEOV2 | 9.77E-11 | -2.4814249 |
| 213988_s_at | SAT1 | 9.97E-11 | 3.8122218 |
